# Real-World Evidence for Breast Cancer Risk Stratification: Insights from the P.I.N.K. Study

**DOI:** 10.3390/cancers18152435

**Published:** 2026-07-29

**Authors:** Michela Franchini, Francesca Denoth, Stefania Pieroni, Francesco Innocenti, Giada Anastasi, Edgardo Montrucchio, Sabrina Molinaro

**Affiliations:** 1 Institute of Clinical Physiology, National Research Council, 56124 Pisa, Italy; francesca.denoth@cnr.it (F.D.); giadaanastasi@cnr.it (G.A.); sabrina.molinaro@cnr.it (S.M.); 2 Department of Biotechnology, Chemistry and Pharmacy, University of Siena, 53100 Siena, Italy; 3 Unit for Analysis, Public Health Agency of Sweden, SE-106 30 Stockholm, Sweden; 4 Senologica Srl, 19124 La Spezia, Italy; emontrucchio@gmail.com

**Keywords:** breast cancer, lifestyle, risk profile, integrated data

## Abstract

**Background/Objectives:** Early detection of breast cancer (BC) substantially improves outcomes, with a 5-year survival rate of 99% for early-stage disease. Risk-based screening, which tailors recommendations according to individual clinical, personal, and lifestyle factors, is a promising strategy to enhance early detection. However, previous studies have reported inconsistent evidence of association with BC risk for several modifiable lifestyle factors including premenopausal adiposity, sedentary behaviour, oral contraceptive use. To address these uncertainties, we investigated the association between clinical, personal, and modifiable lifestyle factors and BC risk in the Prevention, Imaging, Network, Knowledge (P.I.N.K.) project. P.I.N.K. generated real-world evidence by integrating clinical data with information collected through a structured questionnaire evaluating the associations between BC and clinical, personal, and lifestyle factors in 27,123 Italian women aged ≥ 40 years, who underwent integrated breast imaging diagnostics. **Methods:** Characteristics of women with and without BC were compared. Adjusted logistic regression, Least Absolute Shrinkage and Selection Operator (LASSO), and Random Forest models were applied to identify relevant predictors. Variables selected by at least two methods were included in a multivariate logistic regression model, with BC diagnosis as the outcome. **Results:** Accordingly with previous studies a consistent association was found for high breast density, late menopause, overweight, abdominal adiposity, cardiovascular-cerebrovascular and oncological comorbidities and smoking. Greater consumption of plant-based foods, lower intake of red and processed meat, and regular physical activity were associated with lower odds of BC as well. Conversely, an unexpected inverse association was assessed for menstrual irregularities, hyperlipidaemia, and oral contraceptive use. **Conclusions:** The integration of multiple sources of information both clinical and self-reported data collected directly from women adds to the growing evidence on the role of modifiable lifestyle factors in BC risk in supporting more targeted prevention strategies. Future prospective studies should further investigate the long-term effects of lifetime physical activity trajectories, different oral contraceptive formulations, and detailed reproductive history, including menstrual cycle characteristics and menopausal timing, to refine individualized BC risk prediction.

## Simple Summary

Early detection of breast cancer greatly improves outcomes, with the 5-year survival rate reaching 99% when the disease is found at an early stage. One promising approach to improve early detection is risk-based breast screening, which tailors screening recommendations according to a woman’s individual risk factors, including medical history, personal characteristics, and lifestyle habits. The Prevention, Imaging, Network, Knowledge (P.I.N.K.) study was created to collect real-world data from a large group of women across Italy and to support the development of more personalized breast cancer screening strategies. By combining information gathered during routine breast clinic visits with answers provided by women through a structured questionnaire, we examined factors associated with the development of breast cancer. Our findings identified several personal characteristics that were more strongly linked to breast cancer occurrence, highlighting the value of integrating clinical and self-reported information to better understand individual risk and support more targeted prevention and screening efforts. By providing additional evidence on the contribution of potentially modifiable risk factors, this study helps identify priorities for future research aimed at improving breast cancer risk stratification, informing the development of more targeted screening strategies, and facilitating discussions with women about the impact of lifestyle on breast cancer risk.

## 1. Introduction

Breast cancer (BC) is by far the most frequently diagnosed cancer among women worldwide. In 2022 an estimated 2.3 million women were diagnosed with BC resulting in 670,000 deaths globally [1].

The American Cancer Society reports that when BC is detected early and remains confined to its original location, the 5-year relative survival rate exceeds 99% [2].

In most countries, BC screening is implemented through age-based, population-wide programs that have been shown to reduce mortality; however, this approach does not consider the substantial heterogeneity in individual risk profiles among women [3]. Differences in individual risk can result in screening approaches that misclassify patients—potentially causing false positives, overdiagnosis, and unnecessary treatment in women at low risk, while still missing interval cancers in high-risk women [4]. Risk-stratified breast screening is an emerging strategy in which decisions regarding the initiation of screening, its frequency, and the choice of imaging modality (including mammography, tomosynthesis, ultrasound, magnetic resonance imaging, and contrast-enhanced mammography) are individualized based on a precise assessment of a woman’s BC risk. The balance between potential benefits and harms of this approach is currently under active investigation [4,5].

The European Collaborative on Personalized Early Detection and Prevention of BC (ENVISION), in its consensus statement, reviewed the landscape of BC risk prediction, as well as approaches to risk-adapted prevention and early detection, including how these can be put into practice. It also outlines key priorities to drive progress across each of these areas [6].

Ongoing clinical trials such as MyPeBS [7], the WISDOM [8] and the PERSPECTIVE I&I project [9] are evaluating the acceptability and practicality of risk-based cancer assessment and screening, as well as their efficiency, health system readiness for implementation, and the related ethical, psychological, socioeconomic, and legal implications. In particular, the WISDOM trial directly compared standard annual mammography with a personalized, risk-based screening approach informed by validated models incorporating age, lifestyle, medical history, and breast density, and included population-wide genetic testing regardless of family history [8].

Despite their potential benefits, implementing risk-based screening strategies in routine practice remains complex, and several important challenges must be addressed before they can be adopted at the population level. It must also be considered that, although several modifiable lifestyle factors have been investigated in previous studies, evidence regarding their contribution to BC risk remains statistically inconsistent for some exposures [10], limiting their integration into personalized risk assessment models.

In particular we refer to the association with BC risk for premenopausal anthropometric measurements, oral contraceptive (OC) use and dietary habits.

To help bridge this gap, the Prevention, Imaging, Network, Knowledge (P.I.N.K.) study was designed to generate real-world evidence from a large cohort of women in Italy, that supports clinical decision-making in early BC detection and informs regulatory policy.

Our previous work comprises three studies based on convenience cohorts of Italian women enrolled in the P.I.N.K. study. The first study described the P.I.N.K. design and presented its initial findings [11]. The second applied a combined model-driven and data-driven approach, to identify specific combinations of characteristics and lifestyle habits associated with an increased risk of BC occurrence among 5601 postmenopausal women [12]. The third study investigated the effect of alcohol consumption on the anticipation of breast lesion diagnosis among 3774 women who contributed at least 4 years to the study [13]. In this context, the aim of this study is to evaluate the association between BC onset and key clinical, personal, and lifestyle factors in a large cohort of premenopausal and postmenopausal women enrolled in the P.I.N.K. study.

By providing additional evidence on the contribution of potentially modifiable risk factors, this study aims to identify priorities for future research focused on improving BC risk stratification, informing the development of targeted screening strategies, and supporting discussions with women about the impact of lifestyle factors on BC risk.

## 2. Materials and Methods

### 2.1. The Ongoing P.I.N.K. Study: Data, Ethical Considerations and Study Population

The P.I.N.K. study coordinated by the Italian National Research Council (CNR) and co-funded by the Umberto Veronesi Foundation, is an ongoing longitudinal multicentric study, started in October 2017, aiming to recruit women aged 40 years and older, presenting spontaneously for routine breast examination at sixteen public or private diagnostic centers across Italy.

The study was submitted and approved by the ethical committees of each participating center. The main approval was provided by the Regional Ethics Committee for Clinical Trials of the Tuscany Region CEAVNO (Prot n 9047 on 19 February 2018), responsible for the University Hospital of Pisa. The study is being conducted in line with the principles set out in the original Declaration of Helsinki and later amendments. Informed consent has been obtained by all participants and data are handled accordingly.

As of March 2025, a dynamic cohort of 27,123 women had participated in the study. The only inclusion criteria were age (older than 40 years) and provision of informed consent to share personal data collected during routine breast examinations. Exclusion criteria included the presence of breast implants, pregnancy, or breastfeeding.

Study participants completed a structured self-administered questionnaire comprised of four sections: (a) sociodemographic characteristics (marital status, educational level, and employment status); (b) medical and reproductive history (age at menarche and menopause, hormonal therapy, oral contraceptive use, pregnancy, breastfeeding, and assisted reproduction); (c) lifestyle factors, including dietary habits, physical activity, smoking, and alcohol consumption; and (d) family history, documenting the occurrence of cancer among first-degree relatives.

In addition, to evaluate their BC risk or diagnosis each woman received a routine clinic examination through an integrated breast imaging diagnostic including a mammogram (MX) and at least a second imaging test among ultrasound (US or Automated Breast Volume Scanner—ABVS) and/or tomosynthesis (DBT) and/or magnetic resonance (MR) and/or contrast-enhanced spectral mammography (CEM) in the same visit.

Relevant BC diagnostic data were provided by radiologists and included clinical breast examination findings, MX, DBT, US or ABVS, MRI, CEM, cytological and microhistological reports, diagnostic conclusions, and descriptions of cancer cases based on post-surgical histopathological reports. Clinical data collection was standardized through a common data dictionary and a centralized database implemented within a secure web-based platform, which radiologists accessed using personal credentials.

Further details on the study design can be found elsewhere [11].

### 2.2. Statistical Analysis

BC diagnoses detected during the recruitment period (2018–2025) were defined as a binary outcome, coded as 0 for the absence of malignant lesions and 1 for the presence of BC, including both in situ and invasive carcinoma, as confirmed by post-surgical histopathological examinations.

Descriptive analyses were performed by calculating the proportion of women with and without a diagnosis of BC across a set of personal characteristics. Differences in the distributions between the two groups of women were assessed using the Wilcoxon rank-sum test for continuous variables. Associations between categorical variables were evaluated using Pearson’s Chi-squared test.

Based on both self-reported information and data collected by radiologists, each participant was characterized according to three distinct profiles: (1) non-modifiable factors (Profile 1), (2) personal and medical history features (Profile 2), and (3) modifiable lifestyle habits (Profile 3).

Among all the risk factors included in each profile, a subset of features was selected through a stability selection procedure to identify the variables that provide the most informative contribution to explaining the occurrence of BC in women [14,15,16].

Initial multivariable logistic regression models were fitted to quantify the association between each risk factor and the outcome (dichotomized BC diagnosis), reporting adjusted odds ratios. Each base model was adjusted for three covariates, namely age at diagnosis, educational level, and type of diagnostic center (dichotomized). P.I.N.K. centers, including both public and private clinics, were dichotomized as 0 for first-line centers (territorial diagnostic facilities) and 1 for second-line centers (specialized, higher-level, or referral diagnostic facilities) to control for the potential effect of the diagnostic ability to detect BCs.

Educational level and type of diagnostic center were included as proxies for the propensity to undergo periodic breast examinations on a voluntary basis, a characteristic that may distinguish the convenience sample included in the present study from the female population participating in the national free BC screening program.

To address collinearity and high dimensionality, a penalized logistic regression (Least Absolute Shrinkage and Selection Operator—LASSO) was applied. LASSO performs variable selection by shrinking some coefficients to zero, thereby improving model interpretability and reducing overfitting [17].

Two hundred bootstrap resamples were performed, and in each iteration the model was fitted using L1 regularization. The tuning parameter λ was selected using the “1 standard error” rule (λ.1se). For each variable, selection frequency and the distribution of non-zero coefficients were estimated.

In parallel, a Random Forest (RF) model was fitted using 200 independent bootstrap samples to evaluate potential non-linear relationships and interactions. For each predictor, variable importance and its variability across bootstrap samples were calculated.

Prior to model fitting, a data quality criterion was applied by excluding variables with fewer than 30 observations in at least one outcome stratum. Final predictor selection was based on the convergence of results across the three methods (logistic regression, LASSO, and RF), classifying variables as:

- Class A (strong evidence): selected by ≥2 methods;
- Class B (moderate evidence): selected by 1 method;
- Class C (weak evidence): no convergent evidence.

Class A predictors included non-modifiable factors, personal history characteristics, and modifiable lifestyle-related risk factors. Non-modifiable factors comprised age at the diagnosis or last visit, MX breast density, menopausal status, and age at menopause. MX breast density was classified by radiologists according to the four Breast Imaging Reporting and Data System (BI-RADS) density categories, with BI-RADS A indicating predominantly fatty (least dense) breast tissue.

Information on menopausal status and age at menopause was combined into a derived variable to capture the potential effect of atypical menopausal timing. Because age at menopause cannot be defined for women who had not yet undergone menopause, these women were grouped with those who experienced menopause at the typical age (45–55 years), forming the reference category. Early (21–44 years) and late (>55 years) menopause were retained as separate categories because they represent deviations from the expected timing of menopause and correspond to shorter or longer reproductive lifespans, which may influence cumulative endogenous estrogen exposure and BC risk.

Class A personal history variables included educational level (primary/secondary education, high school, university/postgraduate education), occupational status (employed, unemployed, student, homemaker, retired, or unable to work), and marital status (married/in a civil union, separated/divorced, single, or widowed). Additional clinical information comprised self-reported history (Yes/No) of BC, breast biopsy, history of other cancers, metabolic disorders, hyperlipidemia, menstrual irregularities, cardiovascular or cerebrovascular disease, and hypertension. The presence of breast symptoms at the time of BC diagnosis or at the last clinical visit (Yes/No) was also recorded by the radiologists.

Class A modifiable lifestyle-related risk factors included self-reported smoking habits (never, former, or current smokers categorized as <1, 1–5, 6–10, or >10 cigarettes/day), duration of hormonal contraceptive use (never or <1 year, 1–5 years, or >5 years), body mass index (BMI; underweight, normal weight, overweight, or obese), and waist-to-hip ratio (WHR; waist circumference smaller than, equal to, or greater than hip circumference). BMI, as a marker of overall fat accumulation, was used as a proxy for general obesity, whereas WHR, reflecting abdominal or visceral adiposity, was used as a proxy for central obesity.

Lifestyle factors also included reported adherence to the World Cancer Research Fund (WCRF) recommendations scored by women using a five-point Likert scale and categorized as low, medium, or high/very high, with a score of 3 indicating medium adherence. WCRF recommendations promote regular physical activity, a predominantly plant-based diet, and limited consumption of energy-dense foods, red meat, and salt.

Variables classified as class B and C were not included in the final analysis.

After iterative evaluation, a final logistic regression model was fitted using dichotomized BC diagnosis as the dependent variable, with class A variables included as independent predictors. The model was adjusted for age at diagnosis, educational level, and type of diagnostic center. The final model was subsequently stratified by symptoms at diagnosis, type of center and educational level (Figures S1–S3 respectively).

Results from the final logistic regression model are presented by profile as adjusted odds ratios (ORs) with 95% confidence intervals (CIs). All statistical analyses were conducted using R software (version 4.4.2; R Core Team, Vienna, Austria, 2024). A *p*-value < 0.05 was considered statistically significant.

## 3. Results

### 3.1. Descriptive Analysis

As shown in Table 1, Table 2 and Table 3, between 2018 and 2025, 685 out of 27,123 women in the P.I.N.K. cohort were diagnosed with BC, comprising 84.4% invasive, 15.0% in situ, and 0.6% tumors with both invasive and in situ components. The distribution of molecular subtypes was consistent with that reported in the Italian population, with Luminal A being the most prevalent (51.0%), followed by Luminal B/HER2− (24.0%), Luminal B/HER2+ (11.0%), Triple-Negative (9.0%) and HER2-Enriched (5.0%).

Referring to integrated imaging screenings, the 2018–2025 overall detection rate (DR) was 12.04 per 1000 visits (95% CI: 11.18–12.97). Among asymptomatic women, the DR was 8.22 per 1000 visits (95% CI: 7.51–9.00). The overall DR for mammography was 8.72 per 1000 visits (95% CI: 7.98–9.52), and 5.65 per 1000 visits (95% CI: 5.06–6.30) among asymptomatic women.

Table 1, Table 2 and Table 3 reported the most relevant variables (class A) in each profile.

**Table 1:** Distribution of Class A non-modifiable risk factors between BC patients and non-cancer women.

Non-Modifiable Factors	*N*	Without BC *N* = 26,438	With BC *N* = 685	*p*-Value
Age at BC diagnosis or last visit ^1^	27,123	54 (48; 62)	57 (50; 68)	<0.001 ^3^
Breast density ^2^	26,011			
Bi-Rads A extremely fatty tissue		3239 (13%)	59 (9.3%)	0.066 ^4^
Bi-Rads B		8651 (34%)	230 (36%)	
Bi-Rads C		9367 (37%)	244 (39%)	
Bi-Rads D extremely dense tissue		4121 (16%)	100 (16%)	
Unknown		1060	52	
Menopausal timing ^2^	25,680			<0.001 ^4^
Not in menopause or Regular menopause		22,823 (91%)	551 (88%)	
Early menopause		1391 (5.6%)	35 (5.6%)	
Late menopause		841 (3.4%)	39 (6.2%)	
Unknown		1383	60	

^1^ Median (Q1; Q3); ^2^ *N* (%); ^3^ Wilcoxon rank sum test; ^4^ Pearson’s Chi-squared test.

**Table 2:** Distribution of Class A personal and medical history features between BC patients and non-cancer women.

Personal History Features	*N*	Without BC *N* = 26,438	With BC *N* = 685	*p*-Value
Educational qualification ^1^	26,815			<0.001 ^2^
Primary or secondary education		4115 (16%)	173 (26%)	
High school graduation		12,278 (47%)	290 (44%)	
University or postgraduate degree		9764 (37%)	195 (30%)	
Unknown		281	27	
Marital status ^1^	26,804			<0.001 ^2^
Married/In a civil union		18,191 (70%)	416 (63%)	
Separated/Divorced		2971 (11%)	86 (13%)	
Single		3801 (15%)	92 (14%)	
Widow		1182 (4.5%)	65 (9.9%)	
Unknown		293	26	
Occupational status ^1^	25,935			<0.001 ^2^
Employed		17,400 (69%)	350 (55%)	
Seeking employment		525 (2.1%)	10 (1.6%)	
Student		52 (0.2%)	2 (0.3%)	
Housewife		4653 (18%)	174 (27%)	
Retired		2616 (10%)	97 (15%)	
Unable to work		55 (0.2%)	1 (0.2%)	
Unknown		1137	51	
History of breast cancer ^1^	27,123			<0.0013 ^3^
No		24,220 (92%)	500 (73%)	
Yes		2218 (8.4%)	185 (27%)	
Breast Biopsy ^1^	26,092			<0.0013 ^3^
No		18,648 (73%)	340 (55%)	
Yes		6823 (27%)	281 (45%)	
Unknown		967	64	
History of other cancers ^1^	27,123			<0.0013 ^3^
No		24,971 (94%)	619 (90%)	
Yes		1467 (5.5%)	66 (9.6%)	
Metabolic disorders ^1^	23,346			0.0183 ^3^
No		9098 (40%)	250 (45%)	
Yes		13,691 (60%)	307 (55%)	
Unknown		3649	128	
Hyperlipidemia ^1^	23,194			0.53 ^3^
No		14,627 (65%)	353 (63%)	
Yes		8009 (35%)	205 (37%)	
Unknown		3802	127	
Menstrual irregularities ^1^	21,949			<0.0013 ^3^
No		14,812 (69%)	404 (77%)	
Yes		6611 (31%)	122 (23%)	
Unknown		5015	159	
Cardiovasc. and cerebrovascular diseases ^1^	22,835			<0.0013 ^3^
No		16,004 (72%)	339 (60%)	
Yes		6267 (28%)	225 (40%)	
Unknown		4167	121	
Hypertension ^1^	22,947			<0.0013 ^3^
No		17,449 (78%)	374 (66%)	
Yes		4932 (22%)	192 (34%)	
Unknown		4057	119	
Symptomatology at BC diagnosis/last visit ^1^	27,123			<0.001 ^3^
asymptomatic		26,179 (99%)	463 (68%)	
symptomatic		259 (1.0%)	222 (32%)	

^1^ *N* (%); ^2^ Pearson’s Chi-squared test; ^3^ Wilcoxon rank sum test.

**Table 3:** Distribution of Class A modifiable lifestyle factors between BC patients and non-cancer women.

Modifiable and Lifestyle Factors	*N*	Without BC *N* = 26,438	With BC *N* = 685	*p*-Value
BMI ^1^	25,596			<0.001 ^2^
Underweight (<18.5)		1292 (5.2%)	31 (5.0%)	
Normal weight (18.5–24.9)		17,196 (69%)	371 (60%)	
Overweight (25–29.9)		4884 (20%)	162 (26%)	
Obese (≥30)		1608 (6.4%)	52 (8.4%)	
Unknown		1458	69	
Waist-to-hip circumference ^1^	25,276			<0.001 ^2^
Narrower		19,120 (78%)	426 (70%)	
Equal		3773 (15%)	115 (19%)	
Wider		1777 (7.2%)	65 (11%)	
Unknown		1768	79	
Smoking habits ^1^	13,013			<0.001 ^2^
<1 cigarette/day		2641 (21%)	39 (13%)	
1–5 cigarettes/day		4406 (35%)	98 (32%)	
6–10 cigarettes/day		2703 (21%)	77 (25%)	
>10 cigarettes/day		2958 (23%)	91 (30%)	
Unknown		13,730	380	
Oral contraceptive use ^1^	26,079			<0.001 ^2^
never or <1 year		11,769 (46%)	370 (59%)	
1–5 years		6662 (26%)	131 (21%)	
>5 years		7020 (28%)	127 (20%)	
Unknown		987	57	
WCRFs compliance:				
1. Stay daily physically active ^1^	25,094			0.012 ^2^
low		9206 (38%)	234 (40%)	
medium		8941 (36%)	234 (40%)	
high/very high		6358 (26%)	121 (21%)	
Unknown		1933	96	
2. Follow a plant-based diet ^1^	25,041			<0.0012
low		3963 (16%)	122 (21%)	
medium		10,266 (42%)	261 (45%)	
high/very high		10,226 (42%)	203 (35%)	
Unknown		1983	99	
3. Follow a varied diet ^1^	25,358			0.8 ^2^
low		1997 (8.1%)	46 (7.6%)	
medium		8839 (36%)	214 (35%)	
high/very high		13,913 (56%)	349 (57%)	
Unknown		1689	76	
4. Limit red/cured meats ^1^	25,019			0.002 ^2^
low		4397 (18%)	134 (23%)	
medium		9534 (39%)	235 (40%)	
high/very high		10,501 (43%)	218 (37%)	
Unknown		2006	98	
5. Limit salt consumption ^1^	25,069			0.10 ^2^
low		3635 (15%)	105 (17%)	
medium		8445 (35%)	216 (36%)	
high/very high		12,386 (51%)	282 (47%)	
Unknown		1972	82	

^1^ *N* (%); ^2^ Pearson’s Chi-squared test.

#### 3.1.1. Non-Modifiable Factors

The distributions of age at BC diagnosis or last clinical visit and menopausal timing differed between women with and without BC. Late menopause, defined as menopause occurring after 55 years of age, was more prevalent among women with BC (Table 1).

#### 3.1.2. Personal and Medical History Features

Most personal history features reported by women showed statistically significant differences between women with and without BC. Lower educational levels, widowhood, and voluntary or involuntary unemployment were more frequently observed among women with BC, as well as a clinical history of comorbidities, except for metabolic disorders and menstrual irregularities (Table 2).

#### 3.1.3. Modifiable Lifestyle Factors

Increased body composition was positively associated with BC occurrence, as were smoking habits and low adherence to several WCRF recommendations, including regular physical activity, plant-based diet consumption, and limited intake of red and processed meats (Table 3). An inverse association was observed between oral contraceptive use and BC occurrence (Table 3).

### 3.2. Final Logistic Regression Analysis

**Figure 1:**
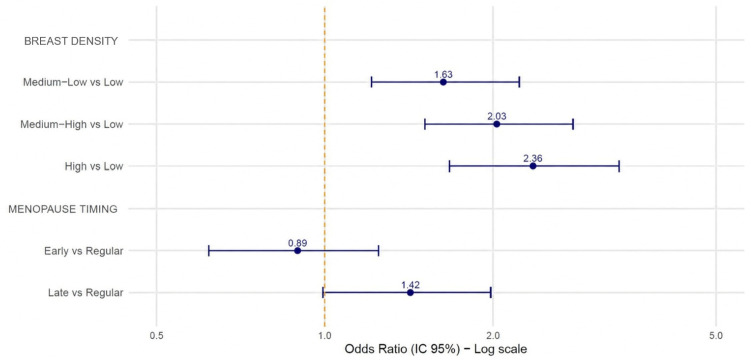
Focus on the association between Class A non-modifiable factors and BC onset.

**Figure 2:**
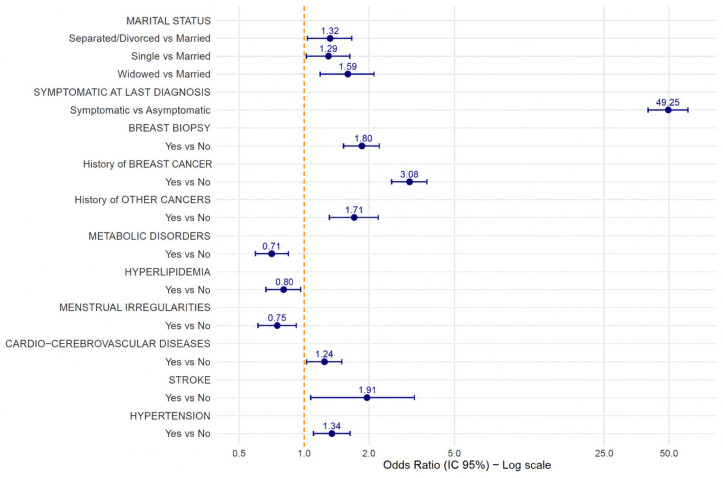
Focus on the association between Class A personal and medical history features and BC onset.

**Figure 3:**
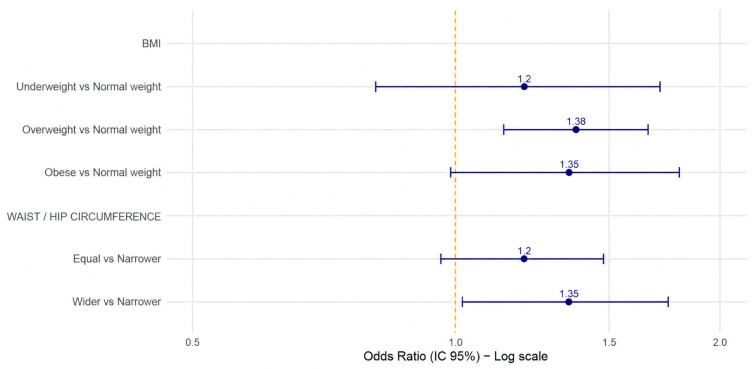
Focus on the association between Class A anthropometric measurements and BC onset.

**Figure 4:**
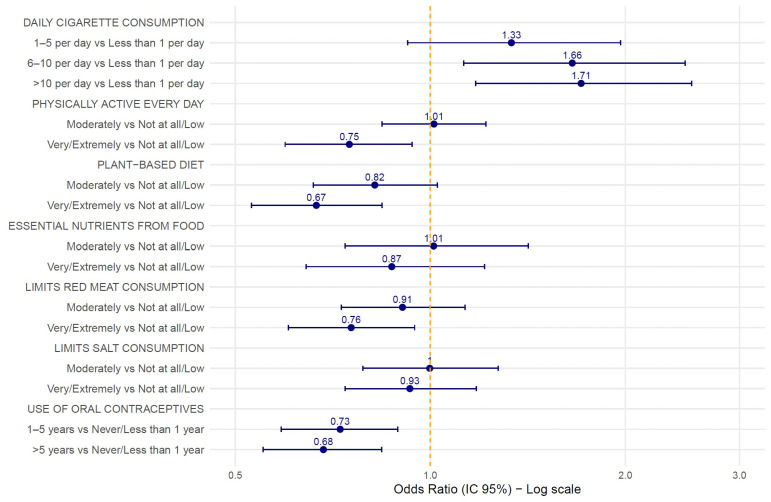
Focus on the association between Class A modifiable factors and BC onset.

Figure 1, Figure 2, Figure 3 and Figure 4 present the adjusted ORs and corresponding CIs for the variables included in the final model (Class A), grouped according to the three profiles.

#### 3.2.1. Non-Modifiable Factors

BC risk increased progressively with breast density (Figure 1). Women with the highest breast density (Bi-rads D) exhibit more than a twofold higher risk compared with those with the lowest breast density (OR: 2.36; 95% CI: 1.67–3.36). This finding was particularly pronounced among women who were asymptomatic at diagnosis (Bi-rads asymptomatic OR: 2.11 95% CI: 1.40–3.21; Bi-rads symptomatic OR: 1.11 95% CI: 0.45–2.78) and among those attending first-line diagnostic centers (Bi-rads first-line center OR: 2.81 95% CI: 1.83–4.39; Bi-rads second-line center OR: 1.71 95% CI: 0.96–2.78), as shown in Figures S1 and S2.

Late menopause was associated with an increased risk of BC (OR: 1.42; 95% CI: 0.99–1.98) compared with regular menopausal timing (Figure 1), particularly among women presenting asymptomatic at the diagnosis (Late menopause asymptomatic OR: 1.62 95% CI: 1.06–2.38; Late menopause symptomatic OR: 1.0 95% CI: 0.36–2.90) and those attending second-line diagnostic centers (Late menopause second-line center OR: 1.81 95% CI: 1.03–2.99; Late menopause first-line center OR: 1.22 95% CI: 0.75–1.88) as shown in Figures S1 and S2.

#### 3.2.2. Personal and Medical History Features

Unmarried status was significantly associated with BC occurrence, with widowhood showing the highest risk (OR: 1.59; 95% CI: 1.18–2.12). Single (OR: 1.29; 95% CI: 1.02–1.63) and the other unmarried categories (OR: 1.32; 95% CI: 1.04–1.66) showed a comparable level of risk (Figure 2).

As expected, the presence of symptoms at the time of BC diagnosis or at the last clinical visit is highly associated with BC diagnosis (OR: 49.25; 95% CI: 39.75–61.04). The presence of symptoms at the time of BC diagnosis represents a major clinical feature considered by radiologists during diagnostic evaluation. Therefore, the strong association of this variable with BC risk reflects its established role in clinical evaluation rather than representing a novel risk factor.

From a clinical point of view, a history of BC (OR: 3.08; 95% CI: 2.55–3.72), breast biopsy (OR: 1.80; 95% CI: 1.53–2.13), or other cancers (OR: 1.71; 95% CI: 1.31–2.21), as well as cardiovascular or cerebrovascular diseases (OR: 1.24; 95% CI: 1.02–1.49), in particular stroke (OR: 1.91; 95% CI: 1.04–3.22) or hypertension (OR: 1.34; 95% CI: 1.1–1.6), were significantly associated with BC onset (Figure 2).

An inverse association was observed between suffering or having suffered from metabolic disorders (OR: 0.71; 95% CI: 0.60–0.84) or hyperlipidaemia (OR: 0.80; 95% CI: 0.67–0.97). An inverse association was also observed with a history of menstrual irregularities (OR: 0.75; 95% CI: 0.61–0.92), which may reflect less consistent lifetime exposure to oestrogens, a key determinant of BC risk [16]. Among women younger than 50 years, the proportion reporting a history of menstrual irregularities was 9% higher in those without cancer. All these inverse associations were more pronounced among women who were asymptomatic at diagnosis, as shown in Figure S1.

#### 3.2.3. Modifiable Lifestyle Factors

Our results showed that being overweight (BMI ≥25 and <30; OR: 1.38; 95% CI: 1.14–1.67) and presenting abdominal adiposity (waist circumference greater than hip circumference—OR: 1.35; 95% CI: 1.02–1.76) were both associated with an increased risk of BC (Figure 3), in particular among women with the lowest educational level (BMI ≥25 and <30; OR: 2.48; 95% CI: 1.05–5.16; waist circumference greater than hip circumference—OR: 1.81; 95% CI: 1.16–2.76) and among those who were asymptomatic at diagnosis (BMI ≥25 and <30; OR: 1.44; 95% CI: 1.14–1.80; waist circumference greater than hip circumference—OR: 1.47; 95% CI: 1.05–2.01) as shown in Figures S1 and S3.

Furthermore, greater adherence to the WCRF recommendations—specifically engaging in daily physical activity (OR: 0.76; 95% CI: 0.60–0.94), consuming plant-based foods (OR: 0.67; 95% CI: 0.53–0.84), and limiting red and processed meat intake (OR: 0.76; 95% CI: 0.60–0.95)—was associated with a lower risk of BC (Figure 4).

Conversely, BC risk increased progressively with the intensity of cigarette smoking. Smoking 6–10 cigarettes per day was associated with a 66% increased risk (OR: 1.66; 95% CI: 1.13–2.47), while smoking more than 10 cigarettes per day was associated with a 71% increased risk (OR: 1.71; 95% CI: 1.18–2.53). Risk from cigarette smoking is particularly relevant among women with the lowest and the highest educational levels and presented asymptomatic at the visit Figures S1 and S3.

Finally, BC decreased with increasing duration of oral contraceptive (OC) use, with the longest duration of use (more than 5 years) associated with the lowest risk (OR: 0.68; 95% CI: 0.55–0.84), in particular among women with medium or high levels of education (Figure S3).

## 4. Discussion

It is widely proven that women with different personal and medical characteristics and different lifestyles form heterogeneous cohorts that exhibit different levels of BC risk [3]. Current screening paradigms largely follow a one-size-fits-all approach and present several limitations, including the lack of adjustment of screening frequency based on individual risk profiles, unnecessary radiation exposure, frequent recalls, unnecessary surgical referrals, biopsies, false-positive and false-negative results, and overdiagnosis. Projects like the WISDOM Randomized Clinical Trial showed that a specific risk-based screening strategy (using genetic, clinical, and risk-factor information to tailor screening intensity and prevention counseling) was noninferior to annual screening for the prespecified safety outcome of advanced-stage BC [8].

The P.I.N.K study is in line with these statements, as it aimed to select a subset of individual features that provide the most informative contribution to explaining BC occurrence (Class A variables), starting from a large number of clinical data provided by radiologists and self-reported data collected directly from women. The P.I.N.K study involves a convenience cohort of 27,123 women of the age of 40 years and above, presenting spontaneously for routine breast examination at sixteen public or private diagnostic centers across Italy. Women were observed between 2018 and 2025, and 685 women were diagnosed with BC.

Class A variables, all included in the same final adjusted regression model, are grouped in three distinct profiles: non-modifiable factors, personal and medical history features and modifiable lifestyle habits.

Among non-modifiable risk factors, breast density was found to be associated with BC risk. Women with BI-RADS density C had a 2.0-fold increased risk of BC (95% CI: 1.51–2.78), and those with BI-RADS density D had a 2.4-fold increased risk (95% CI: 1.67–3.36) compared with women with BI-RADS density A. These findings are consistent with a recent systematic review and meta-analysis, which reported a 3.89-fold higher BC occurrence (95% CI: 2.47–6.13) among women with BI-RADS density D compared with those with BI-RADS density A [18]. Late menopause, a proxy of longer cumulative endogenous estrogen exposure, was also associated with an increased risk of BC (OR: 1.42; 95% CI: 0.99–1.98), whereas a history of menstrual irregularities, included among the personal and medical history features, appeared to be inversely associated with BC (OR: 0.75; 95% CI: 0.61–0.92). These findings may be interpreted considering studies on menstrual cycle–related patterns. Olsson reported that, compared with healthy women and those with benign breast disease, women who developed BC were more likely to have shorter, regular menstrual cycles and greater lifetime menstrual activity [19]. Consistent with this interpretation, the Collaborative Group on Hormonal Factors in Breast Cancer reported that BC risk increases by approximately 3.5% for each additional year of age at menopause [20]. Evidence suggests that the main proliferative phase of the menstrual cycle occurs during the mid-luteal phase, when elevated progesterone and estrogen levels stimulate epithelial alveolar bud formation through increased mitotic activity, supporting a proliferative role of progesterone. Conversely, mammary gland epithelia regress during the late luteal or the follicular phase when newly formed alveolar buds undergo apoptosis and tissue remodeling occurs to return the mammary gland to its basic architecture [21].

Because variation in menstrual cycle length mainly reflects differences in the follicular phase, shorter cycles and greater lifetime menstrual activity may increase estrogen exposure and epithelial cell proliferation, a recognized prerequisite for carcinogenesis [19,22].

Although reproductive history is a well-established determinant of BC risk, our findings indicate an inverse association between BC incidence and longer duration of OC use. This observation differs from the results of Rana et al., in which six of the seven included studies reported a higher BC risk among ever-users compared to never-users of OCs; however, these associations were not statistically significant in four of the six studies [10]. One plausible biological explanation for our findings involves OC-induced modulation of estrogen physiology. Combined OCs suppress ovarian estradiol production and introduce ethinyl estradiol, which is metabolized differently from endogenous estradiol. This shift can alter the overall profile of circulating estrogen metabolites. Particular attention has been given to hydroxylated estrogen metabolites, especially 4-hydroxyestrone and 16α-hydroxyestrone, which have been implicated in breast carcinogenesis due to their potential genotoxic and proliferative effects. BC tissue has been shown to exhibit elevated levels of 4-hydroxyestrone compared with normal breast tissue, supporting a role for these metabolites in malignant transformation. By partially suppressing endogenous estradiol synthesis and modifying metabolic pathways, OC use may reduce the relative formation or activity of these high-risk metabolites. Furthermore, genetic variation appears to influence this relationship. Specifically, carriers of the T allele of the NAD(P)H:quinone oxidoreductase (NQO1) gene—an enzyme involved in detoxification of quinone intermediates and protection against oxidative DNA damage—demonstrated a reduced BC risk with OC use. This may reflect compensatory upregulation of alternative metabolic or DNA repair pathways in individuals with reduced NQO1 activity, thereby enhancing cellular resilience to estrogen-derived oxidative stress [23,24].

Estradiol levels are also closely related to body composition and may contribute to the mechanisms underlying increased central obesity. Furthermore, the association between overweight and BC risk differs between premenopausal and postmenopausal women, likely reflecting differences in estradiol concentrations. During the premenopausal period, women with overweight or obesity exhibited markedly lower estradiol concentrations compared with those with a normal BMI. After menopause, overweight and obese women show substantially higher estradiol concentrations compared with those with a normal BMI. Excess adiposity alters adipocyte metabolism through the abnormal accumulation of lipids, which can impair mitochondrial function and induce stress in the endoplasmic reticulum. As a result, obesity is frequently associated with a persistent low-grade inflammatory condition known as metainflammation [25]. In addition, excessive visceral adipose tissue (VAT) is characterized by a higher presence of M1 macrophages, which produce and release pro-inflammatory cytokines and adipokines. These factors promote insulin resistance and disrupt insulin signaling pathways, thereby contributing to the development of BC [26]. In contrast, adipose tissue in individuals with normal body weight is typically characterized by the presence of M2 macrophages, which support anti-inflammatory signaling. The biological activity of adipose tissue also varies according to its type (white, brown, or beige) and anatomical location (e.g., visceral, subcutaneous, or perivascular). Consequently, the health risks linked to obesity depend not only on the total amount of body fat but also on its distribution and metabolic properties [25].

While increased BMI as a measure of general adiposity has been associated with a higher risk of postmenopausal BC, WHR has been linked to BC risk regardless of menopausal status. WHR, which reflects abdominal or visceral fat distribution, is more closely related to the metabolic pathways underlying obesity-related BC risk [26]. We observed that both overweight status (BMI ≥25 and <30; OR: 1.38; 95% CI: 1.14–1.67) and abdominal adiposity (waist circumference greater than hip circumference; OR: 1.35; 95% CI: 1.02–1.76) were associated with comparable increases in BC risk. These findings suggest that the relationship between adiposity and BC risk may not be entirely dependent on menopausal status, in line with evidence from some of the studies included in [10].

We also observed that a diagnosis of hyperlipidemia was associated with a reduced risk of BC. This finding is consistent with evidence from a large retrospective longitudinal cohort study that reported a significantly lower risk of BC among patients with hyperlipidemia (OR 0.67; 95% CI 0.49–0.93; *p* < 0.02), 40% reduced BC mortality and improved long-term survival compared with no hyperlipidemia patients [27]. Our findings are also consistent with a case–control study performed using female Medicare beneficiaries’ data from 2007 to 2011, to assess the association between invasive BC and dyslipidemia [28]. Anti-oxidative and anti-inflammatory properties of high-density lipoprotein cholesterol (HDL-C) have been linked to the reduced risk among postmenopausal women. Experimental research indicates that HDL-C may protect against lipid peroxidation by limiting oxidative damage to low-density lipoprotein cholesterol (LDL-C). Higher circulating HDL-C concentrations both promote anti-inflammatory cytokine production and reduce insulin-like growth factor-I (IGF-I), a hormone associated with increased mammographic breast density in postmenopausal women and with processes involved in carcinogenesis [29].

These observations suggest that metabolic characteristics, together with anthropometric measures assessed across the life course, may provide complementary information for BC risk assessment. As such, they warrant further evaluation for inclusion in the development and validation of multifactorial BC risk prediction models, rather than being considered independent determinants of screening recommendations. Diet has been identified as a key factor influencing both circulating lipid levels and the risk of developing BC. Consistently, we found that consuming plant-based foods (OR: 0.67; 95% CI: 0.53–0.84) and limiting red and processed meat intake (OR: 0.82; 95% CI: 0.66–0.95) were both associated with a lower risk of BC. As well, engaging in daily physical activity (OR: 0.75; 95% CI: 0.60–0.94) represents another protective factor acting through various processes. Taken together, these lifestyle habits help regulate lipid profiles by reducing inflammation, strengthening the immune system’s function, improving insulin sensitivity, and increasing protective HDL-C concentrations, which may in turn inhibit tumor proliferation [30]. Our findings are consistent with previous evidence, including our earlier analyses [12], supporting the protective effects of regular physical activity, a plant-based diet, and limited consumption of red and processed meat. Similarly, the recent overview by Rana and colleagues reported that 23 studies showed the association between physical activity and a reduced risk of BC, regardless of the type of activity, menopausal status and anthropometric measurements in particular among postmenopausal women. Moreover, higher physical activity levels at a young age and lifetime propensity to activity were also inversely associated with BC incidence [10]. The effect of an unbalanced diet was also recently reported by Ratajczak-Pawłowska and colleagues [31]. In particular, a meta-analysis by Wu et al. [32] found that a high intake of red meat was associated with a significantly increased risk of BC in a dose–response manner (RR = 1.07; 95% CI: 1.01–1.14). The association was more pronounced for fresh red meat (RR = 1.13; 95% CI: 1.01–1.26) and processed meat (RR = 1.09; 95% CI: 1.02–1.17). Our results on vegetable and fruit consumption are consistent with the systematic review and meta-analysis performed by Farvid et al. [33], which showed that higher fruit and vegetable intake is associated with a reduced risk of BC. This inverse association was evident not only for overall and postmenopausal BC but also for both estrogen- and progesterone-receptor-positive (ER+/PR+) and receptor-negative (ER−/PR−) subtypes.

Among modifiable lifestyle determinants, tobacco smoking remains a key factor influencing BC risk, with risk increasing in a dose-dependent manner as smoking intensity rises (1–6 cigarettes/day OR: 1.66; 95% CI: 1.13–2.47; >10 cigarettes/day OR: 1.71; 95% CI: 1.18–2.53). This is particularly relevant among women with lower or higher educational levels and presenting asymptomatic. This observation aligns with recent epidemiological evidence indicating that women who have ever smoked exhibit a higher risk of BC compared with never smokers independent of menopausal status [10]. Moreover, initiation of smoking prior to first childbirth appears to confer additional susceptibility, likely due to heightened vulnerability of undifferentiated breast tissue [34]. Collectively, these findings support the inclusion of smoking intensity alongside established demographic, reproductive, and anthropometric risk factors in individualized BC risk assessment models. Such an approach may improve the identification of asymptomatic women at elevated risk and inform more personalized prevention strategies, including targeted smoking cessation interventions and, where supported by validated risk prediction tools, risk-adapted screening approaches.

In contrast to previous evidence on the role of alcohol consumption in BC risk [10], including our earlier findings [13], lifetime alcohol exposure was classified as a Class C predictor in the current analysis, reflecting the weaker and less consistent evidence supporting its association with BC (base-adjusted model: OR = 1.06; 95% CI: 0.87–1.31; not selected by either the RF or LASSO models). This discrepancy is likely attributable to differences in the criteria used to define the reference cohort of women included in our previous longitudinal and time-dependent analyses [13]. In those analyses, alcohol consumption emerged as a relevant factor for individualized BC risk assessment, not only because of its established carcinogenic role [10] but also because higher alcohol intake was associated with a significantly faster transition from being free of breast lesions to receiving a breast lesion diagnosis. Moreover, when alcohol consumption was evaluated in combination with smoking status, even moderate alcohol intake approached statistical significance, suggesting a potential interaction between these lifestyle factors [13]. As such, alcohol drinking should be included in the risk assessment models, also considering genetic and sex differences in alcohol metabolism [10].

### Strength and Limitations

The main strength of this study is the large sample size combined with the extensive clinical characterization of participants and diagnoses, supported by detailed radiological imaging data and histopathological findings from biopsy specimens or post-surgical pathological reports. From a methodological perspective, an additional strength lies in the selection of the most informative predictors based on the consistency of findings across complementary model-driven and data-driven approaches, including multivariate logistic regression, RF analysis, and LASSO regression. The convergence of these methods enhances the robustness of predictor selection and reduces the likelihood that the reported associations are driven by the assumptions of a single analytical approach. Nevertheless, several limitations should be acknowledged. First, the observational nature of the study precludes causal inference. Second, lifestyle data were collected using a self-administered paper questionnaire. Although this approach facilitated data collection in a large multicenter cohort, it relied on participants’ understanding and accurate reporting of health-related information, potentially limiting the precision of some variables. In addition, self-reported data are inherently susceptible to recall bias and, for lifestyle-related variables, to social desirability bias, which may have resulted in exposure misclassification. Because such misclassification is likely to be non-differential with respect to disease status, its effect would be expected to attenuate rather than inflate the observed associations.

Third, the generalizability of the findings may be limited by the convenience sampling strategy adopted in the P.I.N.K. study. Compared with the general Italian female population, participants underwent breast imaging within an organized clinical setting and were more likely to have higher educational attainment and to be employed, characteristics that may be associated with greater health literacy, healthier lifestyles, and increased use of preventive healthcare services. Consequently, the prevalence of some lifestyle-related exposures may not be fully representative of the target population, potentially limiting the external validity of the findings. Women who voluntarily participate in screening or observational studies may differ systematically from non-participants with respect to both exposure profiles and health-seeking behaviors, giving rise to self-selection bias. Such women are generally more proactive in managing their health, more likely to adhere to screening recommendations, and potentially more inclined to modify lifestyle behaviors, which may influence both the prevalence of risk factors and the observed associations with BC. To reduce the impact of this potential bias, educational level and type of diagnostic center were included as adjustment variables in the base multivariate models, as proxies for differential access to healthcare services, participation in opportunistic BC screening, and the propensity to engage in preventive health behaviors.

## 5. Conclusions

Assessing the combined effects of modifiable factors on BC risk is challenging due to substantial population heterogeneity and the multidimensional nature of exposure patterns, which require large, well-characterized datasets. The P.I.N.K. study illustrates the potential of integrating these diverse data sources to identify multidimensional risk profiles and capture the joint effects of preventive and predisposing factors. This framework supports the development of collaborative, data-driven strategies for advancing personalized prevention and maximizing public health impact. Since most existing evidence is derived from retrospective studies based on data collected at a single time point and often relies on averaged exposure measures, future research should prioritize long-term prospective investigations. These studies should comprehensively evaluate reproductive history, menstrual cycle characteristics, different oral contraceptive formulations, and lifetime physical activity trajectories, objectively quantified using metabolic equivalents (METs), to further refine individualized BC risk prediction.

## Data Availability

The datasets presented in this article are not readily available because the P.I.N.K. study raw data sharing is not applicable as specified in the informed consent signed by women.

## Supplementary Materials

The following supporting information can be downloaded at: https://www.mdpi.com/article/10.3390/cancers18152435/s1, Figure S1: Association between risk factors and BC onset, stratified by symptomatology; Figure S2: Association between risk factors and BC onset, stratified by type of diagnostic center; Figure S3: Association between risk factors and BC onset, stratified by educational level.

## References

[1] Kim J., Harper A., McCormack V., Sung H., Houssami N., Morgan E., Mutebi M., Garvey G., Soerjomataram I., Fidler-Benaoudia M.M. (2025). Global patterns and trends in breast cancer incidence and mortality across 185 countries. Nat. Med..

[2] American Cancer Society (2026). Breast Cancer Early Detection and Diagnosis. https://www.cancer.org/cancer/types/breast-cancer/screening-tests-and-early-detection.html.

[3] Clift A.K., Dodwell D., Lord S., Petrou S., Brady S.M., Collins G.S., Hippisley-Cox J. (2022). The current status of risk-stratified breast screening. Br. J. Cancer.

[4] Pedersen L.H., Bigaard J., Vejborg I., Kamstrup P.R., Andersen B., Yang X., Ficorella L., Dennis J., Antoniou A.C., Flyger H. (2026). Risk Stratified Breast Cancer Screening: Early Outcomes and Psychological Impact from PRSONAL—A Randomized Clinical Trial. Breast.

[5] (2019). Horizon 2020. EU-TOPIA: Towards Improved Screening for Breast, Cervical and Colorectal Cancer in All of Europe. CORDIS Project Factsheet. https://cordis.europa.eu/project/id/634753/reporting.

[6] Pashayan N., Antoniou A.C., Ivanus U., Easton D.F., French D., Sroczynski G., Hall P., Cuzick J., Evans D.G., Simard J. (2020). Personalized early detection and prevention of breast cancer: ENVISION consensus statement. Nat. Rev. Clin. Oncol..

[7] Roux A., Cholerton R., Sicsic J., Moumjid N., French D.P., Giorgi Rossi P., Balleyguier C., Guindy M., Gilbert F.J., Burrion J.B. (2022). Study protocol comparing the ethical, psychological and socio-economic impact of personalised breast cancer screening to that of standard screening in the MyPeBS randomized clinical trial. BMC Cancer.

[8] Esserman L.J., Fiscalini A.S., Naeim A., Van’t Veer L.J., Kaster A., Scheuner M.T., LaCroix A.Z., Borowsky A.D., Anton-Culver H., Olopade O.I. (2026). Risk-Based vs Annual Breast Cancer Screening: The WISDOM Randomized Clinical Trial. JAMA.

[9] Seung S.-J., Mittmann N., Ante Z., Liu N., Blackmore K.M., Richard E.S., Wong A., Walker M.J., Earle C.C., Simard J. (2024). Evaluating Real World Health System Resource Utilization and Costs for a Risk-Based Breast Cancer Screening Approach in the Canadian PERSPECTIVE Integration and Implementation Project. Cancers.

[10] Rana M.S., Rahman M.M., Baker J., Houssami N., Yu X.Q., Marinovich M.L. (2025). Association of lifestyle factors with breast cancer incidence: An overview of systematic reviews. Chin. J. Cancer Res..

[11] Franchini M., Pieroni S., Montrucchio E., Nori Cucchiari J., Di Maggio C., Cassano E., Di Nubila B., Giuseppetti G.M., Nicolucci A., Scaperrotta G. (2021). The P.I.N.K. Study Approach for Supporting Personalized Risk Assessment and Early Diagnosis of Breast Cancer. Int. J. Environ. Res. Public Health.

[12] Franchini M., Pieroni S., Denoth F., Scalese Urciuoli M., Colasante E., Salvatori M., Anastasi G., Frontignano C.K., Dogliotti E., Vidali S. (2022). Promote Community Engagement in Participatory Research for Improving Breast Cancer Prevention: The P.I.N.K. Study Framework. Cancers.

[13] Cerrai S., Lachi A., Franchini M., Pieroni S., Anastasi G., Scalese M., Odone A., Gallus S., Smits L., Molinaro S. (2025). Alcohol consumption and breast lesions: Targets for risk-based screening in high-risk Italian women. Breast Cancer.

[14] Meinshausen N., Bühlmann P. (2010). Stability selection. J. R. Stat. Soc. Ser. B Stat. Methodol..

[15] Nogueira S., Sechidis K., Brown G. (2017). On the stability of feature selection algorithms. J. Mach. Learn. Res..

[16] Nicodemus K.K. (2011). Letter to the editor: On the stability and ranking of predictors from random forest variable importance measures. Brief. Bioinform..

[17] Tibshirani R. (1996). Regression Shrinkage and Selection via the Lasso. J. R. Stat. Soc. Ser. B.

[18] Bodewes F.T.H., van Asselt A.A., Dorrius M.D., Greuter M.J.W., de Bock G.H. (2022). Mammographic breast density and the risk of breast cancer: A systematic review and meta-analysis. Breast.

[19] Olsson H.L., Olsson M.L. (2020). The Menstrual Cycle and Risk of Breast Cancer: A Review. Front. Oncol..

[20] Collaborative Group on Hormonal Factors in Breast Cancer (2012). Menarche, menopause, and breast cancer risk: Individual participant meta-analysis, including 118,964 women with breast cancer from 117 epidemiological studies. Lancet Oncol..

[21] Atashgaran V., Wrin J., Barry S.C., Dasari P., Ingman W.V. (2016). Dissecting the Biology of Menstrual Cycle-Associated Breast Cancer Risk. Front. Oncol..

[22] Motuma A., Mussa I., Deressa A., Regassa L.D., Birhanu A. (2025). Central obesity increases the risk of breast cancer irrespective of menopausal status in women: Systematic review and meta-analysis. Cancer Treat. Res. Commun..

[23] Fowke J.H., Shu X.-O., Dai Q., Jin F., Cai Q., Gao Y.-T., Zheng W. (2004). Oral Contraceptive Use and Breast Cancer Risk: Modification by NAD(P)H Oxoreductase (NQO1) Genetic Polymorphisms. Cancer Epidemiol. Biomark. Prev..

[24] Santos S.S., Jácome G.P.O., Koifman R., Koifman S. (2014). CYP17, CYP19, and NQO1 genetic polymorphisms and breast cancer susceptibility in young women in Brazil. Br. J. Med. Med. Res..

[25] Kuryłowicz A. (2023). Estrogens in Adipose Tissue Physiology and Obesity-Related Dysfunction. Biomedicines.

[26] Chen H., Yuan M., Quan X., Chen D., Yang J., Zhang C., Nan Y., Luo F., Wan D., Yang G. (2023). The relationship between central obesity and risk of breast cancer: A dose–response meta-analysis of 7,989,315 women. Front. Nutr..

[27] Carter P.R., Uppal H., Chandran S., Bainey K.R., Potluri R., Algorithm for Comorbidities, Associations, Length of Stay and Mortality (ACALM) Research Unit (2017). Patients with a diagnosis of hyperlipidemia have a reduced risk of developing breast cancer and lower mortality rates: A large retrospective longitudinal cohort study from the UK ACALM registry. Eur. Heart J..

[28] Schairer C., Freedman D.M., Gadalla S.M., Pfeiffer R.M. (2018). Lipid-lowering drugs, dyslipidemia, and breast cancer risk in a Medicare population. Breast Cancer Res. Treat..

[29] Ni H., Liu H., Gao R. (2015). Serum lipids and breast cancer risk: A meta-analysis of prospective cohort studies. PLoS ONE.

[30] Pedersen B.K., Saltin B. (2015). Exercise as medicine—Evidence for prescribing exercise as therapy in 26 different chronic diseases. Scand. J. Med. Sci. Sports.

[31] Ratajczak-Pawłowska A.E., Jezierska K., Szymczak-Tomczak A., Zawada A., Rychter A.M., Skoracka K., Dobrowolska A., Krela-Kaźmierczak I. (2025). Lifestyle and Breast Cancer: Prevention and Treatment Support. Cancers.

[32] Wu J., Zeng R., Huang J., Li X., Zhang J., Ho J.C., Zheng Y. (2016). Dietary Protein Sources and Incidence of Breast Cancer: A Dose-Response Meta-Analysis of Prospective Studies. Nutrients.

[33] Farvid M.S., Barnett J.B., Spence N.D. (2021). Fruit and vegetable consumption and incident breast cancer: A systematic review and meta-analysis of prospective studies. Br. J. Cancer.

[34] Bjerkaas E., Parajuli R., Weiderpass E., Engeland A., Maskarinec G., Selmer R., Gram I.T. (2013). Smoking duration before first childbirth: An emerging risk factor for breast cancer? Results from 302,865 Norwegian women. Cancer Causes Control.

