# Supplementary material for: Real-World Evidence for Breast Cancer Risk Stratification: Insights from the P.I.N.K. Study
Source: Cancers (Basel). 2026 Jul 29;18(15):2435. doi: 10.3390/cancers18152435 (PMC13465517; doi:10.3390/cancers18152435)
Supplement: Supplementary file 1 [file cancers-18-02435-s001.zip › cancers-4422397-supplementary.pdf]

Supplementary Materials:

Real-World Evidence for Breast Cancer Risk Stratification: Insights from the P.I.N.K. Study

Michela Franchini [1,2]*, Francesca Denoth[1], Stefania Pieroni[1]*, Francesco Innocenti[1], Giada Anastasi[1], Edgardo Montrucchio[3], Sabrina Molinaro[1] on behalf of the P.I.N.K. Consortium.

1 Institute of Clinical Physiology, National Research Council, 56124 Pisa, Italy; michela.franchini@cnr.it http://orcid.org/0000-0001-5272-341X (M.F.); francesca.denoth@cnr.it (F.D.); stefania.pieroni@cnr.it https://orcid.org/0000-0002-8130-4967 (S.P.); francescoinnocenti@cnr.it (F.I.); giadaanastasi@cnr.it https://orcid.org/0000-0002-5854-3716 (G.A.); sabrina.molinaro@cnr.it (S.M.)

2 Department of Biotechnology, Chemistry and Pharmacy, University of Siena, 53100 Siena, Ital; michela.franchini@cnr.it

3 Senologica Srl, 19124 La Spezia, Italy; emontrucchio@gmail.com (E.M.)

Correspondence: michela.franchini@cnr.it (M.F.); stefania.pieroni@cnr.it (S.P.)

S1 - Association between risk factors and BC onset, stratified by symptomatology

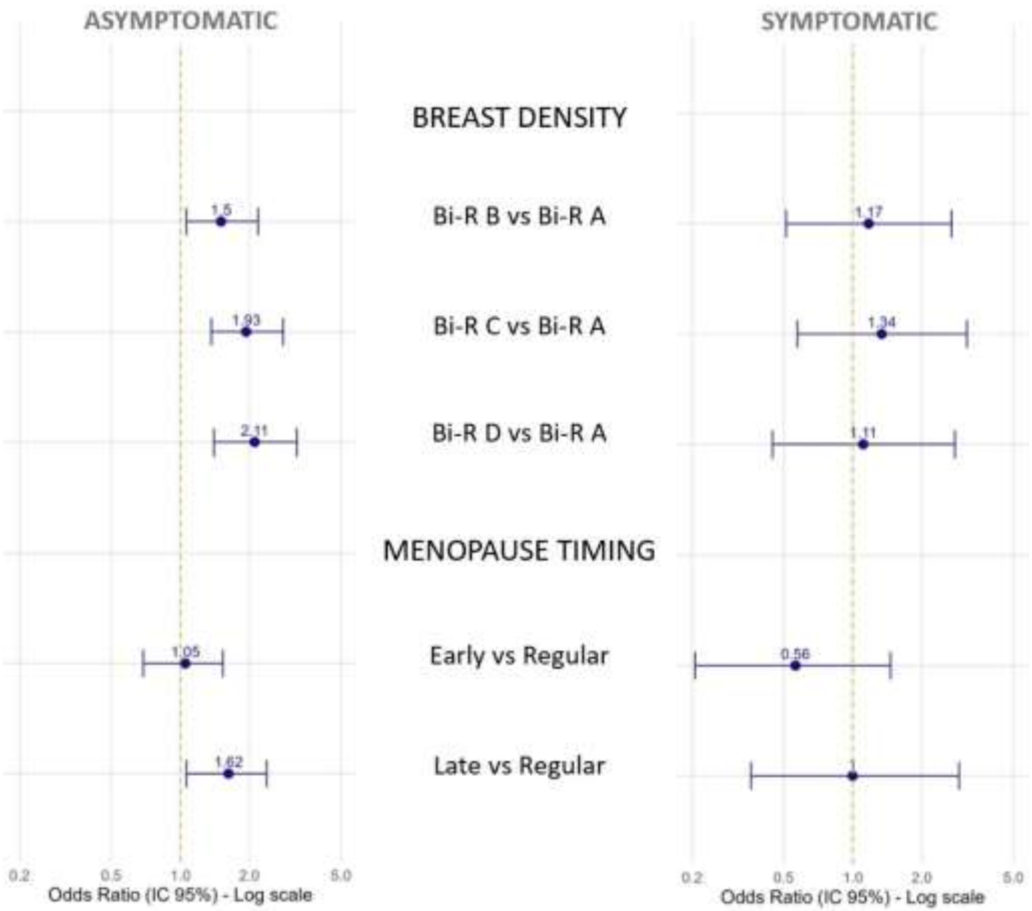

S1a -Non-modifiable factors

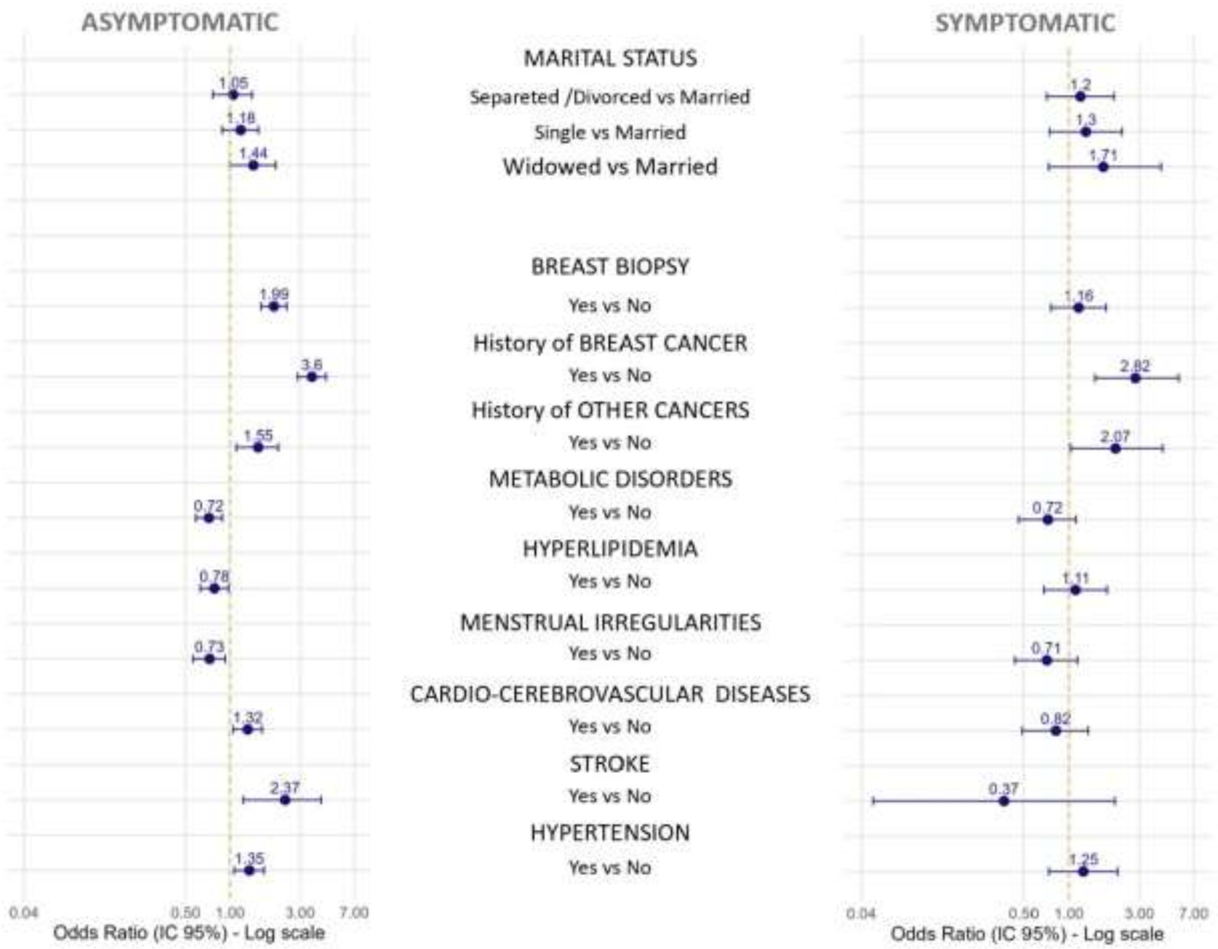

S1b - Personal and medical history features

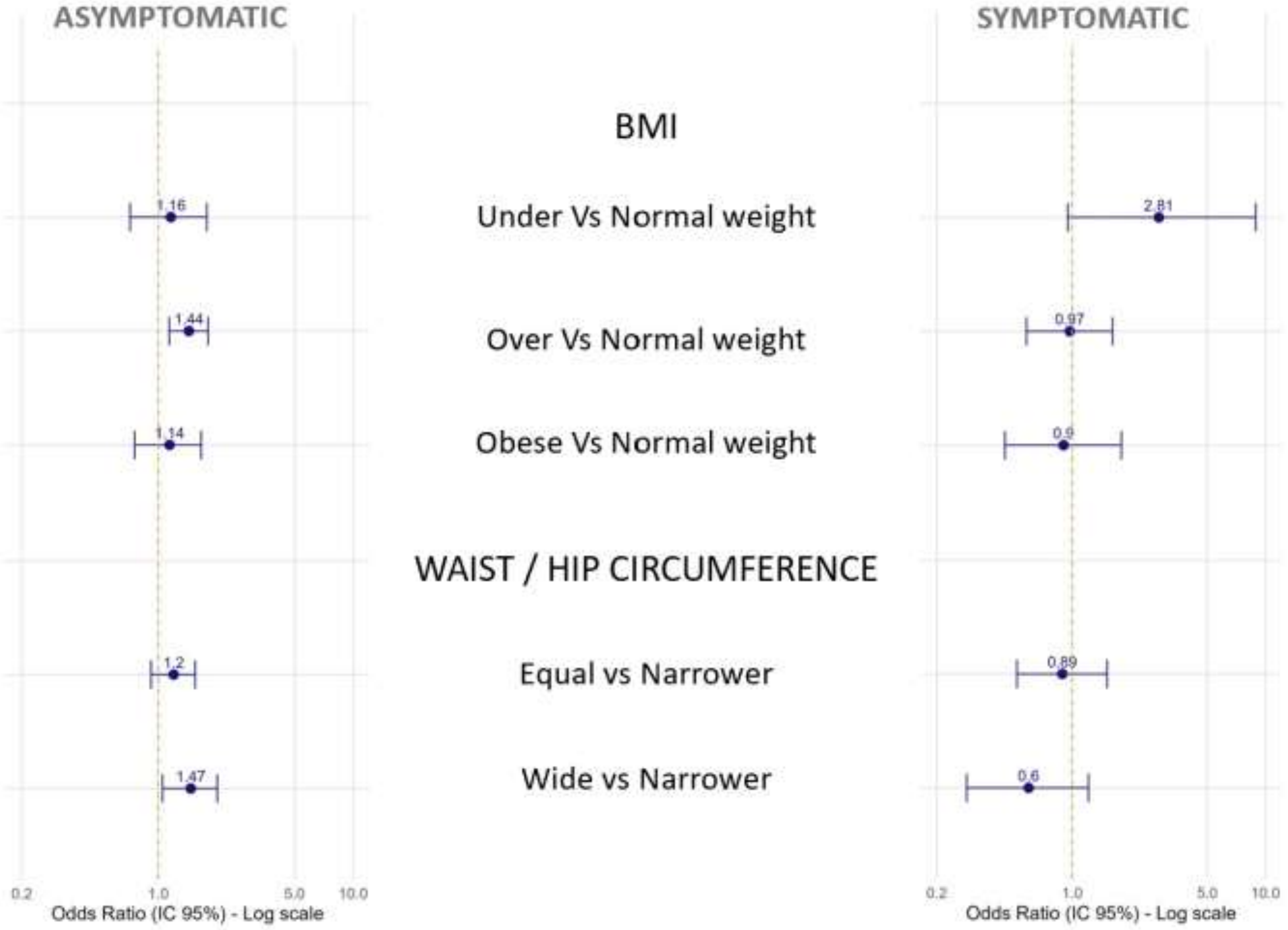

S1c - Anthropometric measurement

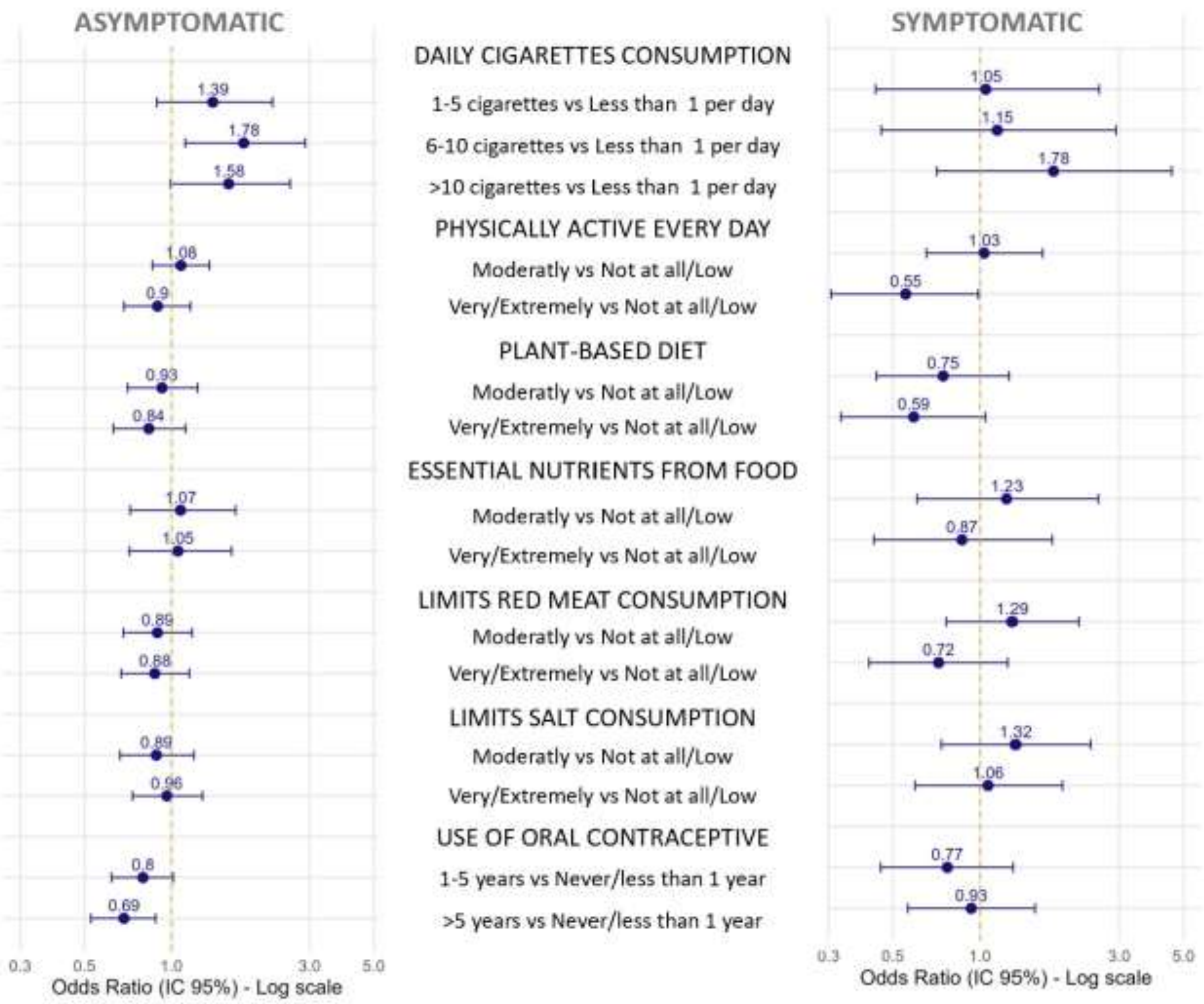

S1d - Modifiable factors

S2 - Association between risk factors and BC onset, stratified by type of diagnostic center

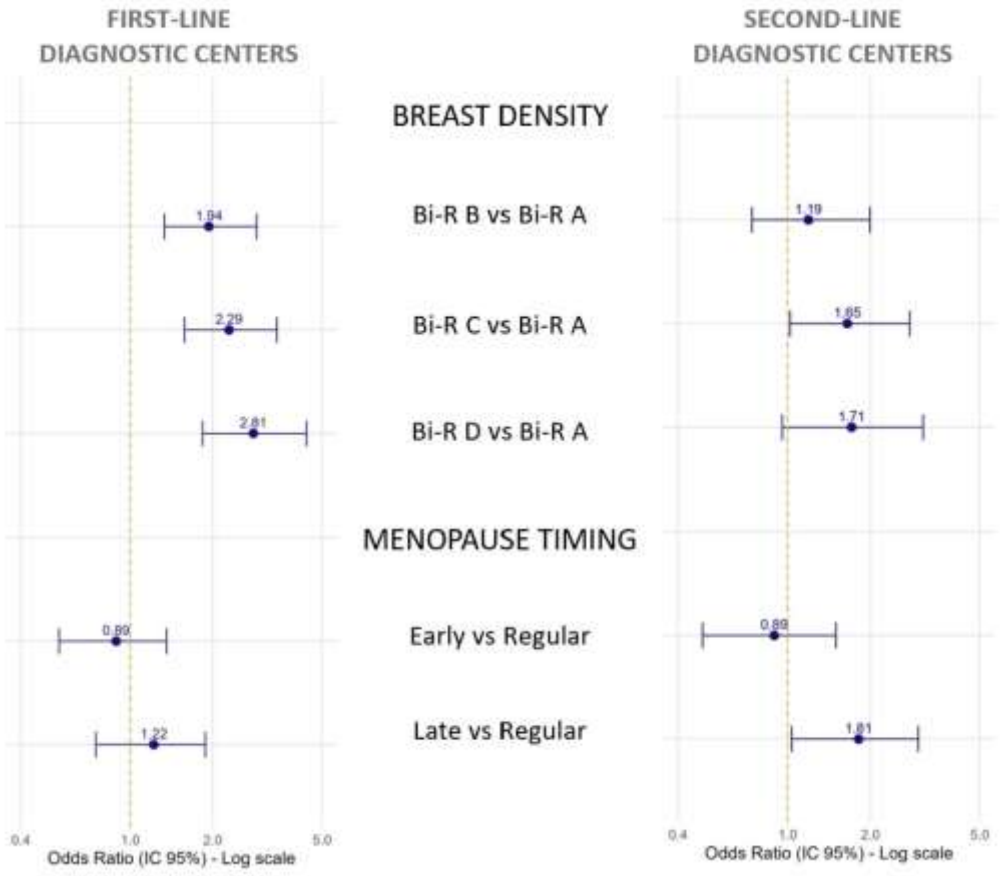

S2a - Non-modifiable factors

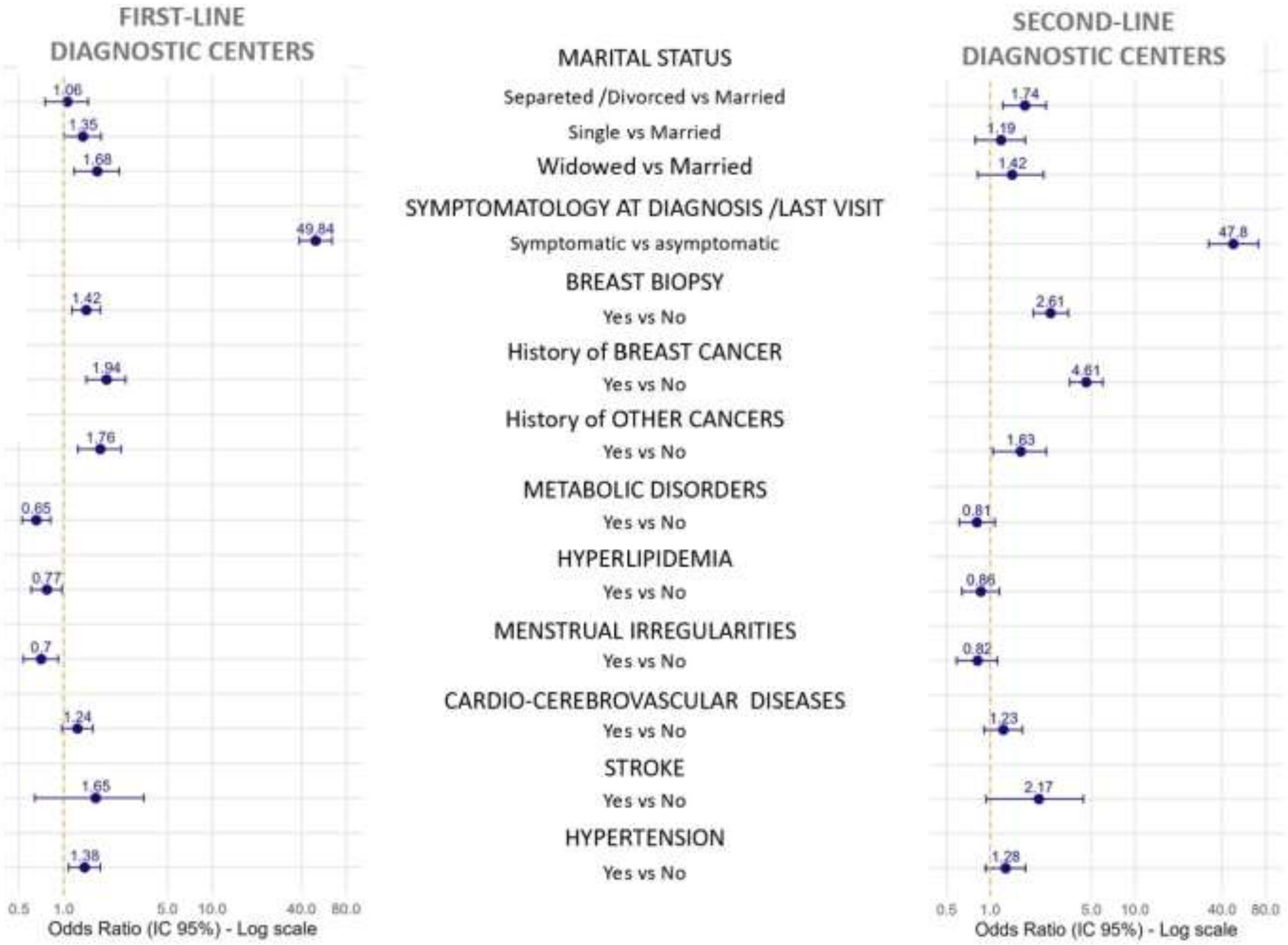

S2b - Personal and medical history features

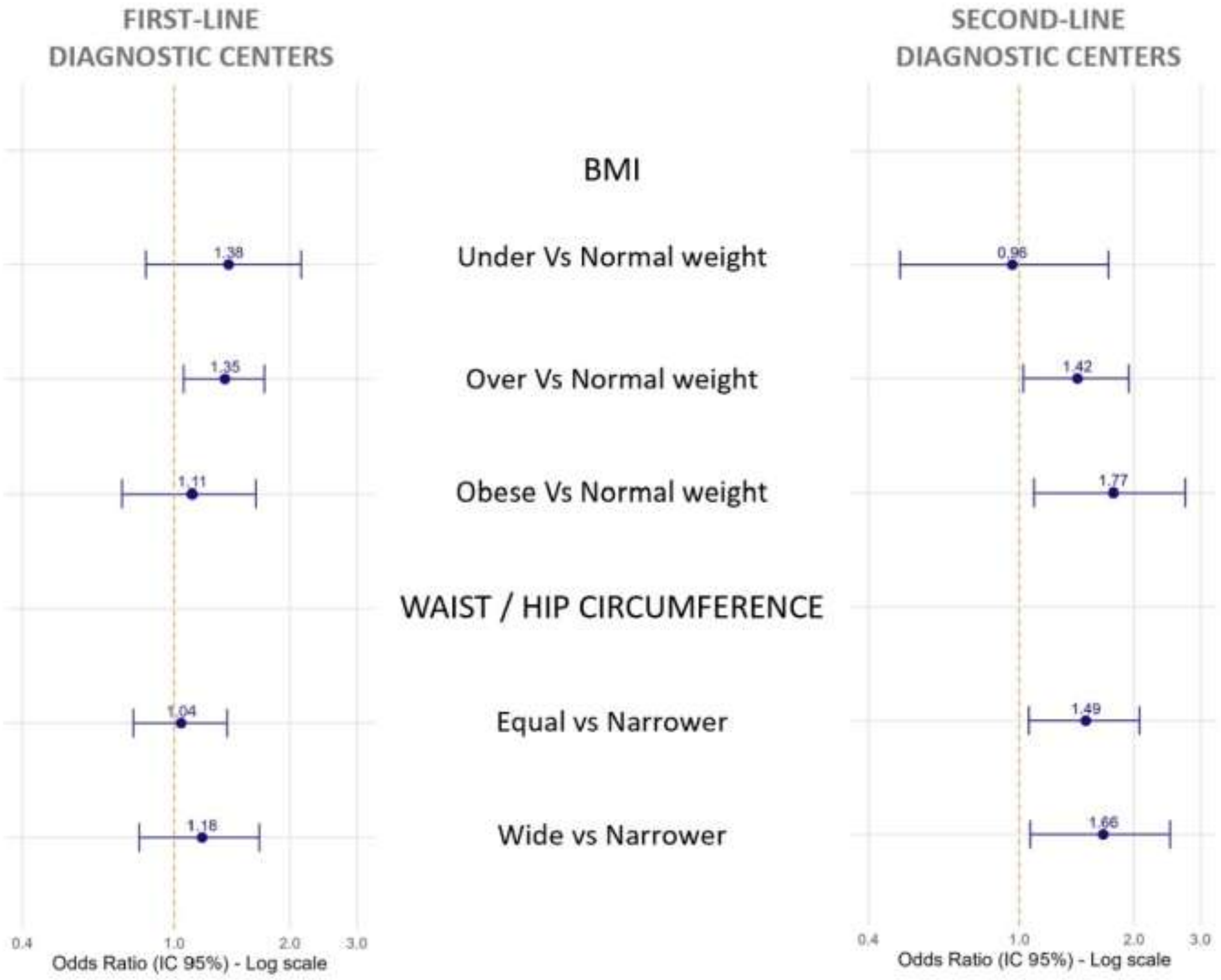

S2c - Anthropometric measurement

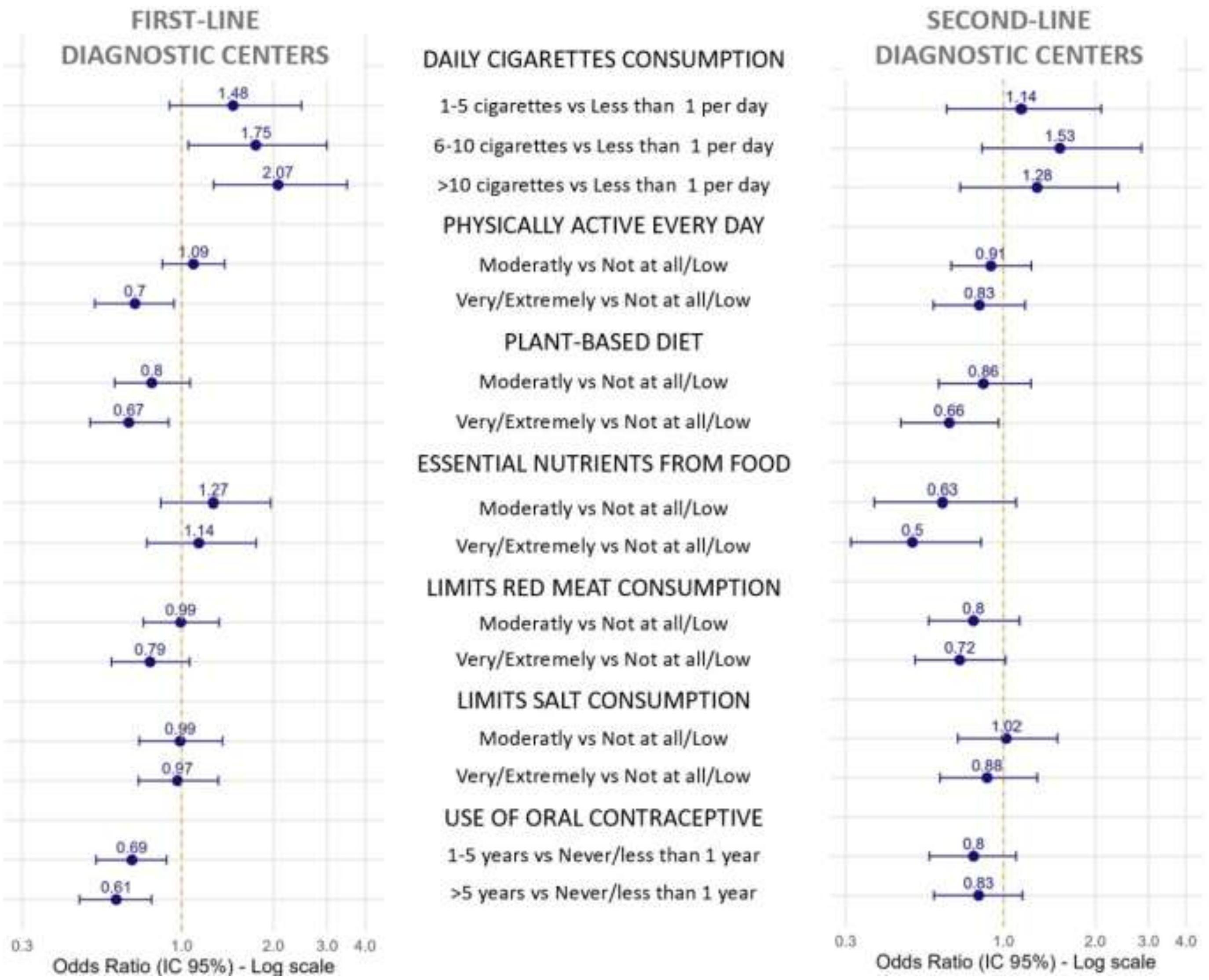

S2d - Modifiable factors

S3 - Association between risk factors and BC onset, stratified by educational level

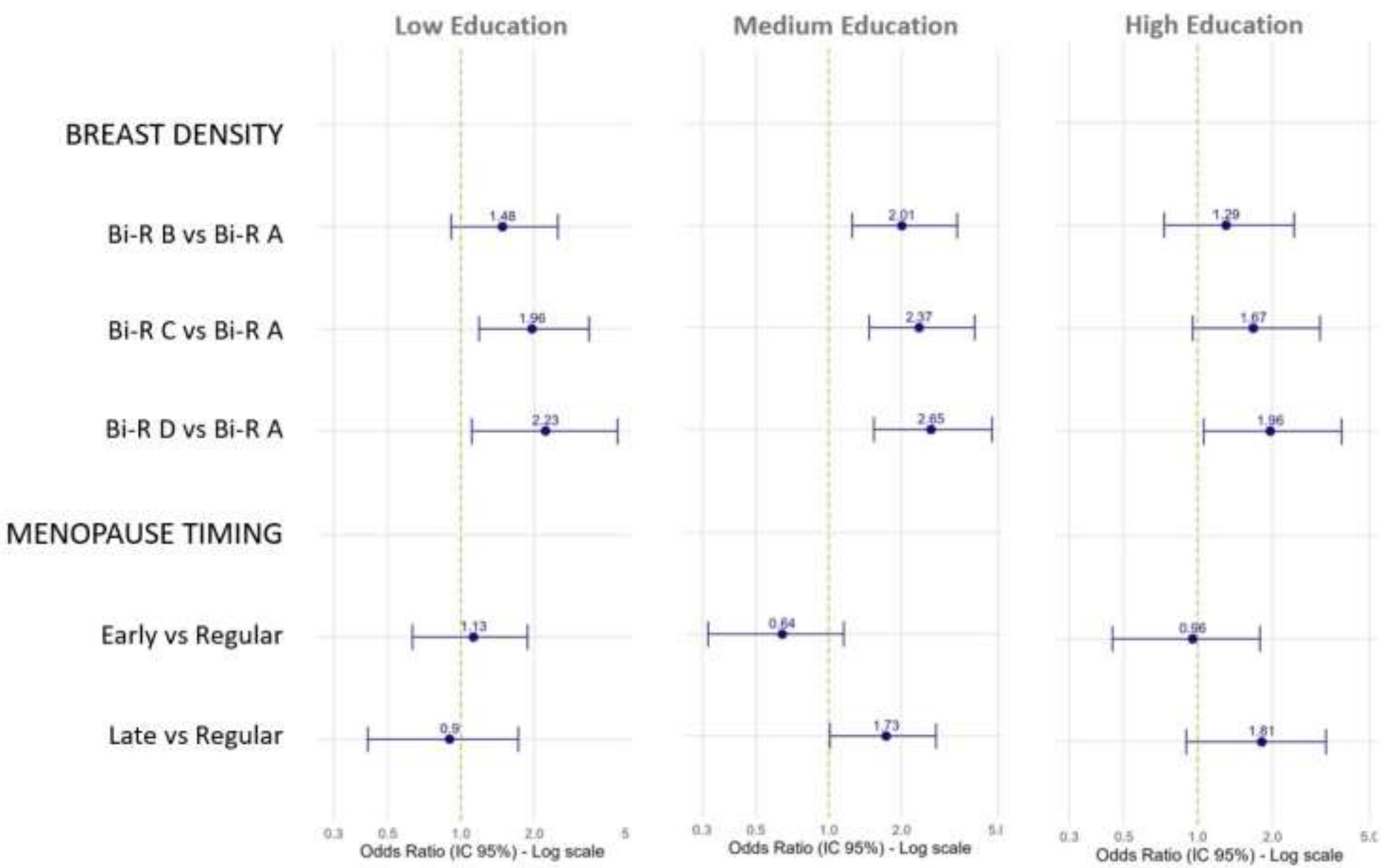

S3a - Non-modifiable factors

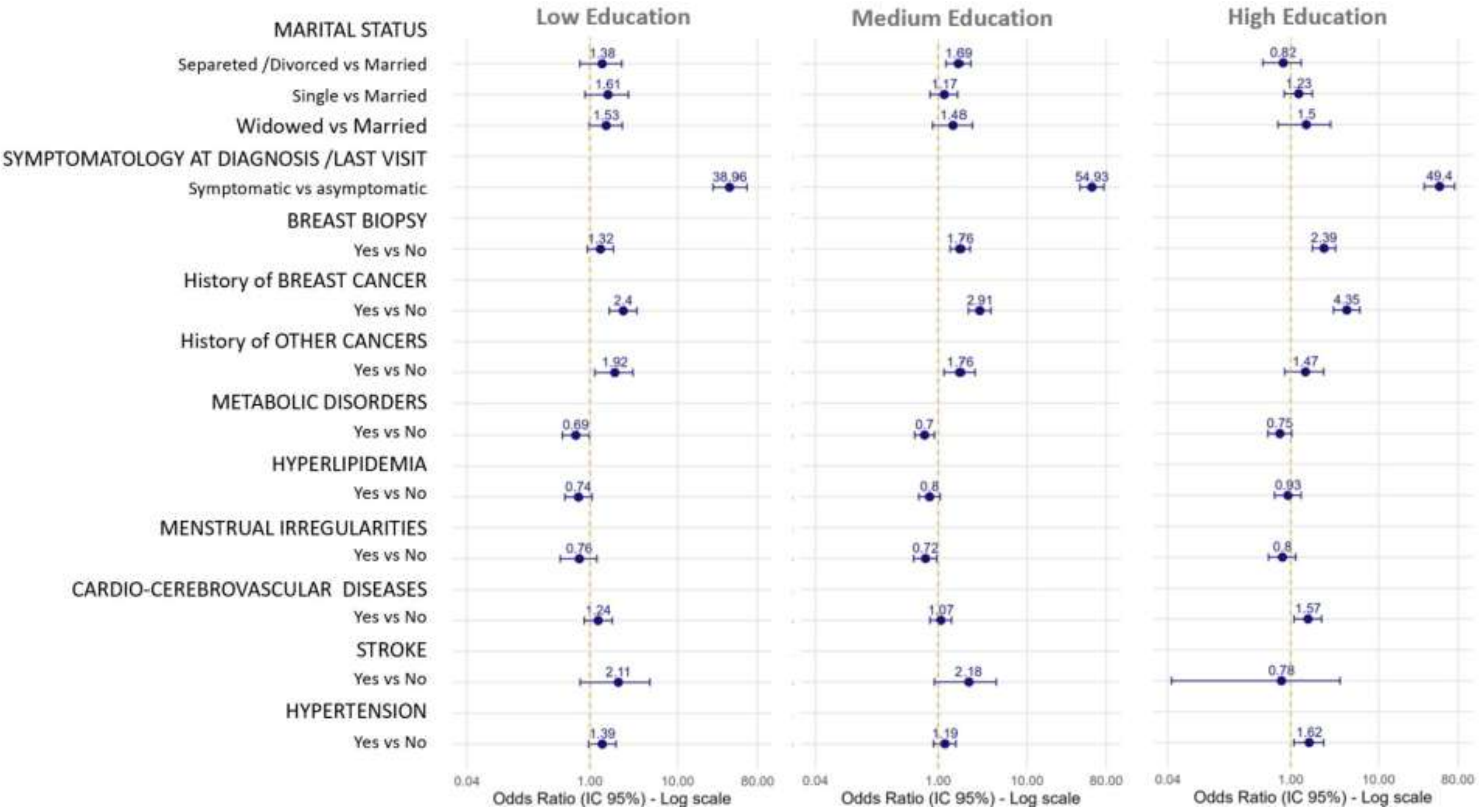

S3b - Personal and medical history features

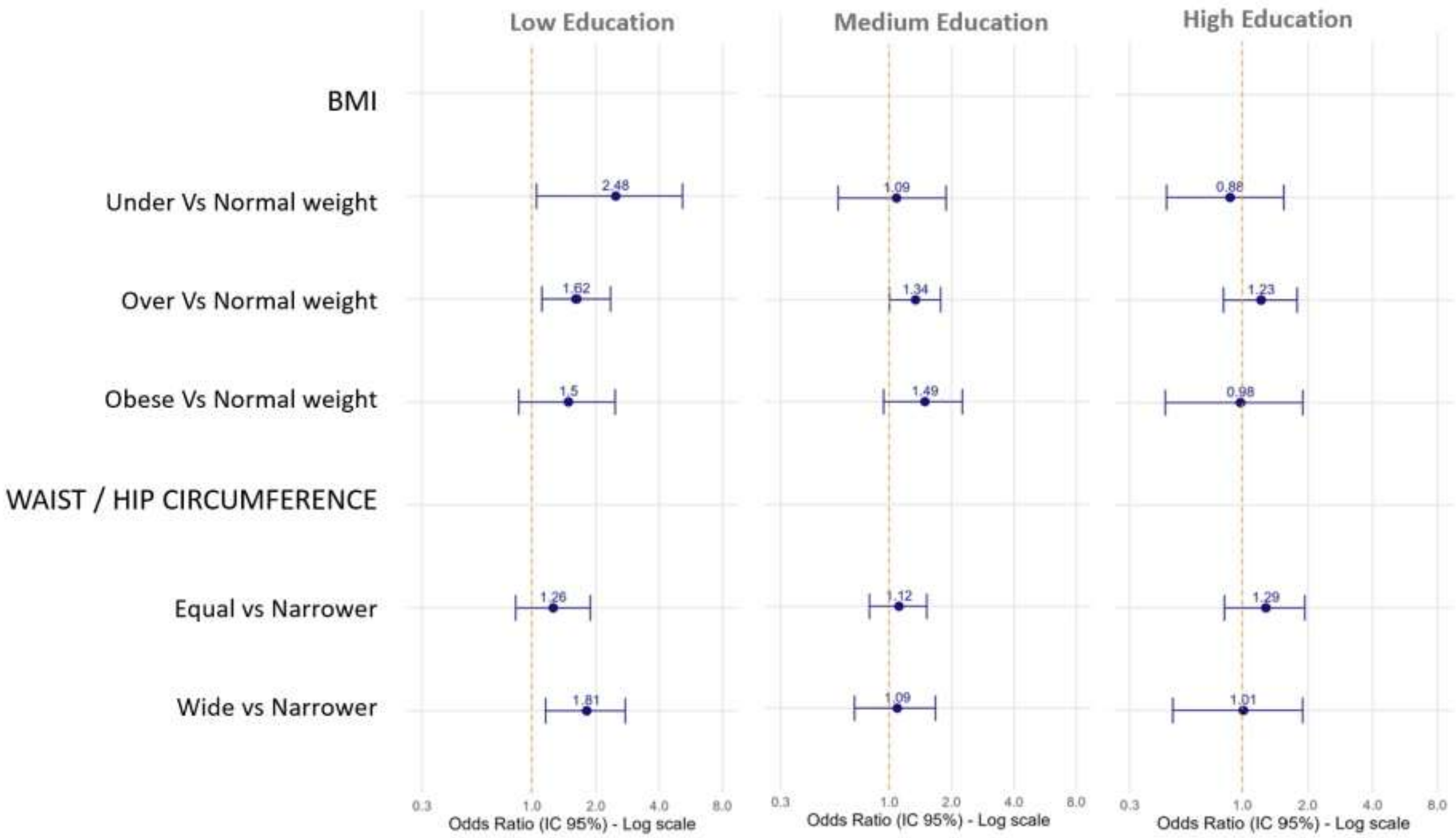

S3c - Anthropometric measurement

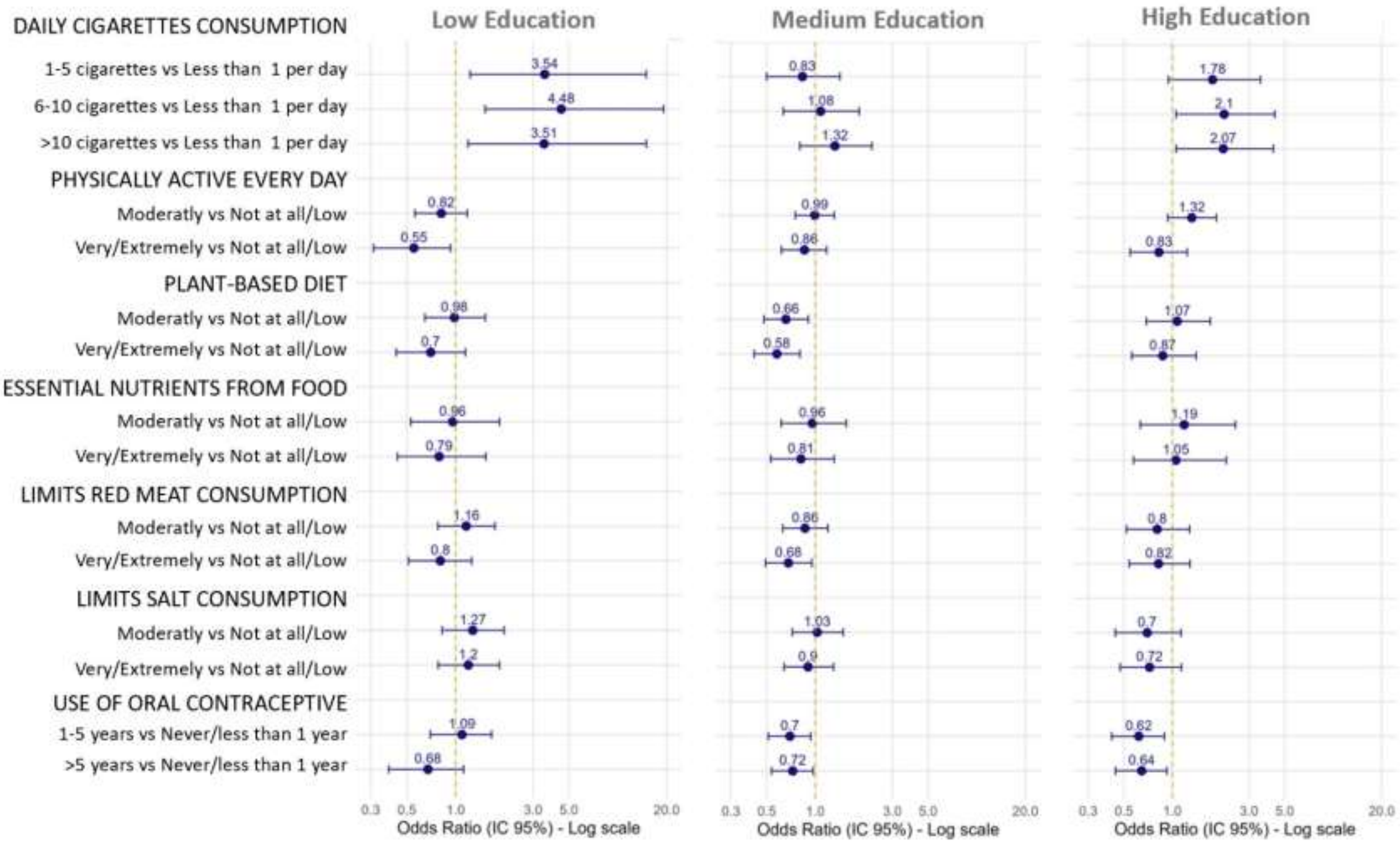

S3d - Modifiable factors
